# A case report of acute promyelocytic leukemia with mycosis fungoides

**DOI:** 10.1097/MD.0000000000036619

**Published:** 2024-01-05

**Authors:** Shasha Dong, Yejing Zhu, Fang Zhang, Yongqin Zhao, Hongjing Zhou

**Affiliations:** a Jining NO. 1 People’s Hospital, Jining, Shandong, China; b Daizhuang Psychiatric Hospital, Jining, Shandong, China.

**Keywords:** acute promyelocytic leukemia, all-trans-retinoicacid, cutaneous T-cell lymphoma, sezary’s syndrome

## Abstract

**Rationale:**

Acute promyelocytic leukaemia (APL) is a rare subtype of acute myelogenous leukaemia. With advances in treatment regimens, namely, introduction of all-trans-retinoicacid, outcomes have drastically improved, its side effects should not be ignored. Mycosis fungoides is one of the side effects of all-trans-retinoicacid treatment, but it may also be a clinical manifestation before disease progression. However, it rarely appears and is easily overlooked. This leads to being easily misled during the treatment process, affecting the treatment plan, and resulting in adverse consequences. Therefore, early identification and judgment can not only provide appropriate treatment options, but also prevent and treat further disease progression.

**Patient concerns:**

The patient was hospitalized for pancytopaenia. After completing the examination, the patient was finally diagnosed with acute promyelocytic leukaemia (acute myelogenous leukaemia-M3). We administered tretinoin and arsenous acid. Evaluation of the treatment effect on the 7th day after chemotherapy showed that the bone marrow morphology showed complete remission. After the second course of chemotherapy, the patient developed red miliary macular papules, which gradually worsened. After completing relevant inspections, Considering that the cases was complicated with skin mycosis fungoides, the patient was treated with budesonide ointment and methylprednisolone as chemotherapy.

**Diagnoses:**

Upon examination, the patient was initially diagnosed with acute promyelocytic leukaemia. Evaluation of the treatment effect on the 7th day after chemotherapy showed that the bone marrow morphology showed complete remission. After the second course of chemotherapy, we discovered the patient was diagnosed with skin mycosis fungoides.

**Interventions:**

Systemic chemotherapy is first given when a patient was diagnosed with acute promyelocytic leukaemia. After the patient happened skin mycosis fungoides, We have adjusted the treatment plan and supplemented it with other treatment plans based on the original chemotherapy, After 2 months of treatment, his condition gradually improved.

**Outcomes:**

All-trans-retinoicacid in the treatment of APL must be given attention because mycosis fungoides should not only be distinguished from infectious diseases but also be further assessed with regard to disease progression and metastasis.

**Lessons:**

Acute promyelocytic leukemia needs to be treated with arsenic trioxide. All-trans-retinoicacid in the treatment of APL must be given attention mycosis fungoides. Early diagnosis can guide accurate treatment, which is of great help in alleviating the pain of patients and improving the cure rate.

## 1. Introduction

Acute promyelocytic leukaemia (APL) is a rare subtype of acute myelogenous leukaemia. APL can manifest in a manner similar to other subtypes of acute myelogenous leukaemia, such as easy fatigue, weakness, infections, and bleeding complications. By the time symptoms develop, the bone marrow of affected patients often has many abnormal promyelocytes. Not only does this contribute to blood cell count abnormalities such as leukopaenia or leukocytosis, anemia, and thrombocytopaenia, but this population of malignant cells can provoke a unique severe coagulopathy.^[1]^ With advances in treatment regimens, namely, introduction of all-trans-retinoicacid (ATRA), outcomes have drastically improved, with complete remission rates approaching 100% in ATRA-based regimens and event-free survival rates recorded as more than 90%.^[2]^ Although the treatment scheme of the new drug has brought a high cure rate, its side effects should not be ignored.

Mycosis fungoides is a malignant tumor of T-cell origin. Mycosis fungoides (MF) and Sezary’s syndrome (SS) are the most common cutaneous T-cell lymphomas, accounting for 2% to 3% of non-Hodgkin lymphomas. MF accounts for 60% of CTCL; SS only accounts for 5%. In the early stage, patients usually show discrete skin lesions similar to eczema or extensive erythema. Patients with advanced disease may have fungal tumors or leukaemia, with eventual involvement of lymph nodes and internal organs. Most MF patients develop disease early, with an inert course, whereas SS is an aggressive erythrodermic leukaemia MF variant characterized by obvious blood system invasion and lymph node enlargement.^[3]^

The patient in this case developed a red miliary maculopapular rash after the second course of chemotherapy for primary APL. It was unclear whether it was a side effect of drugs or further progression of the disease and whether it was necessary to adjust the treatment plan, which prompted our investigation.

## 2. Case presentation

**Figure 1.**
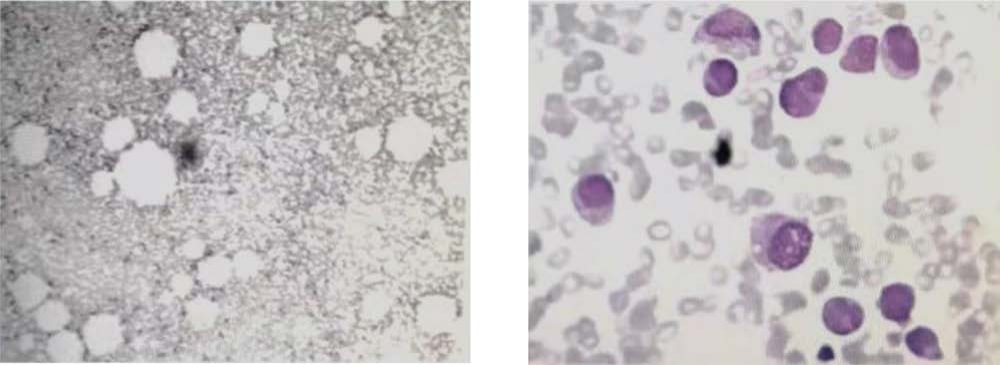
Chemical staining of the bone marrow aspirate of the patient.

**Figure 2.**
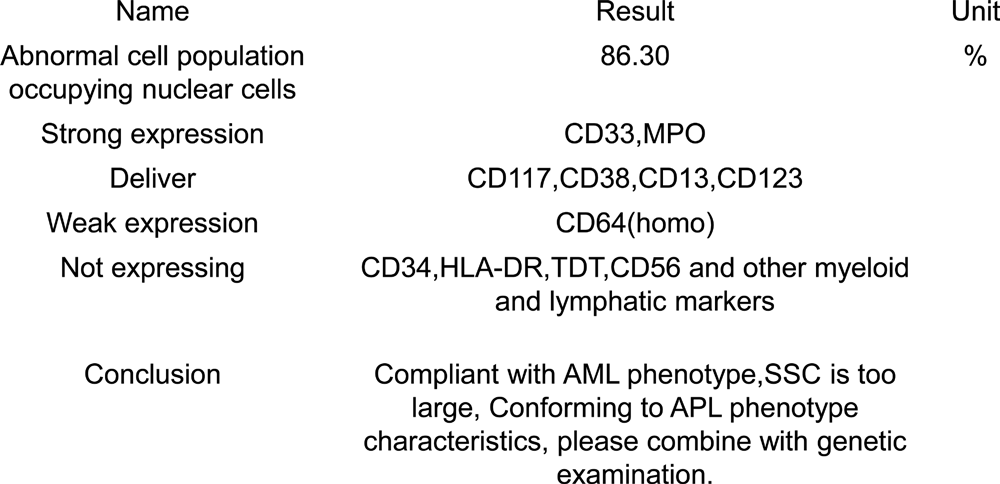
Flow cytometry of the bone marrow aspirate of the patient, including he was in APL. APL = acute promyelocytic leukaemia.

**Figure 3.**
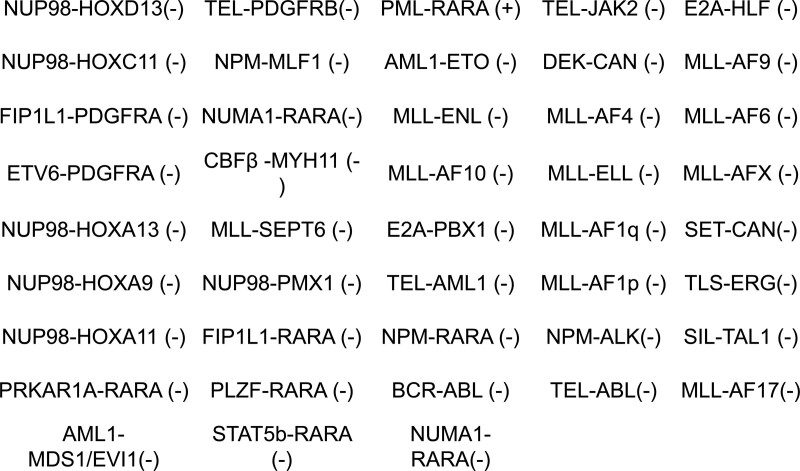
The detection of 43 fusion genes in leukemia patients using real-time fluorescent probe PCR method.

**Figure 4.**
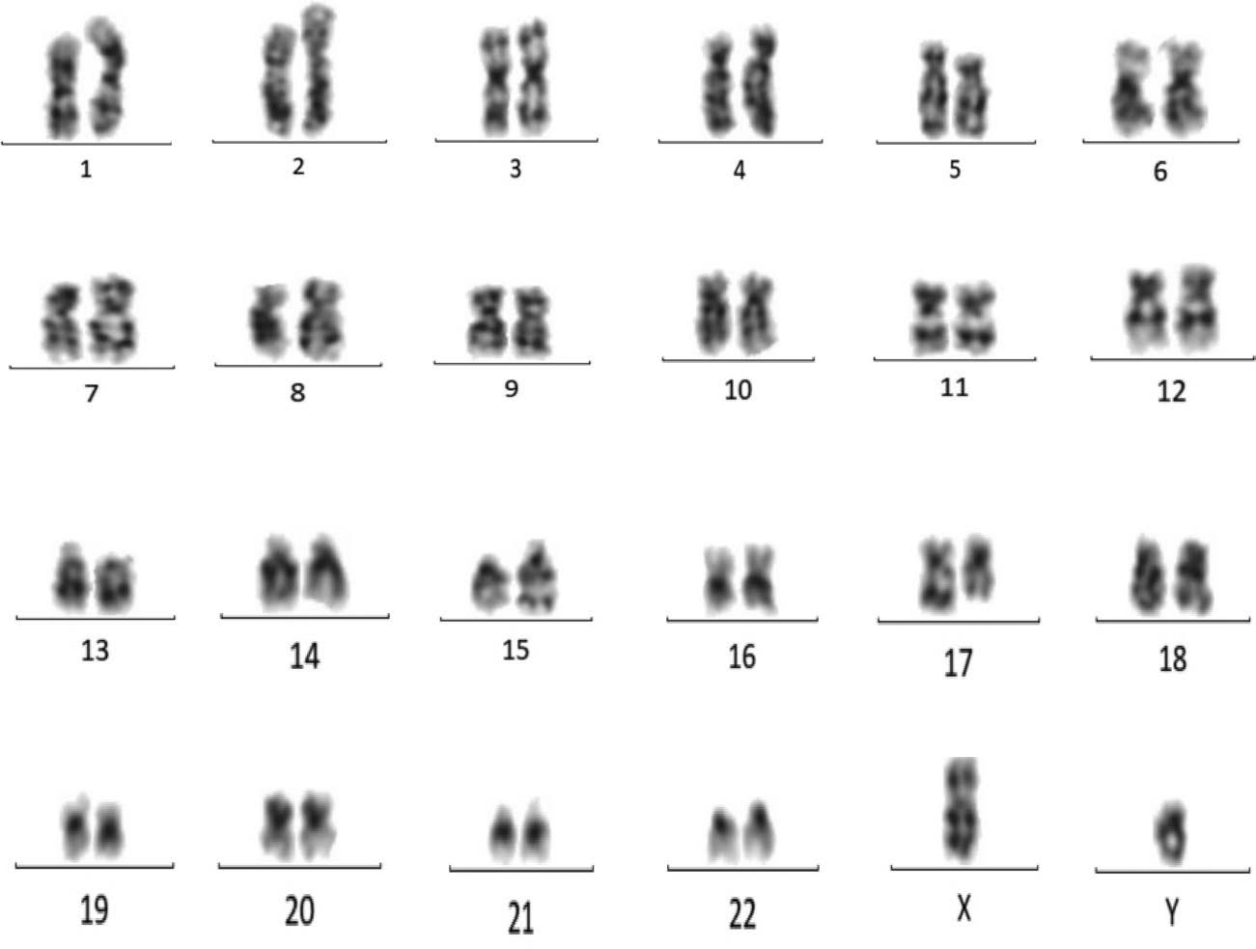
Chromosome karyotype analysis: Visible clonal abnormalities t (15; 17).

**Figure 5.**
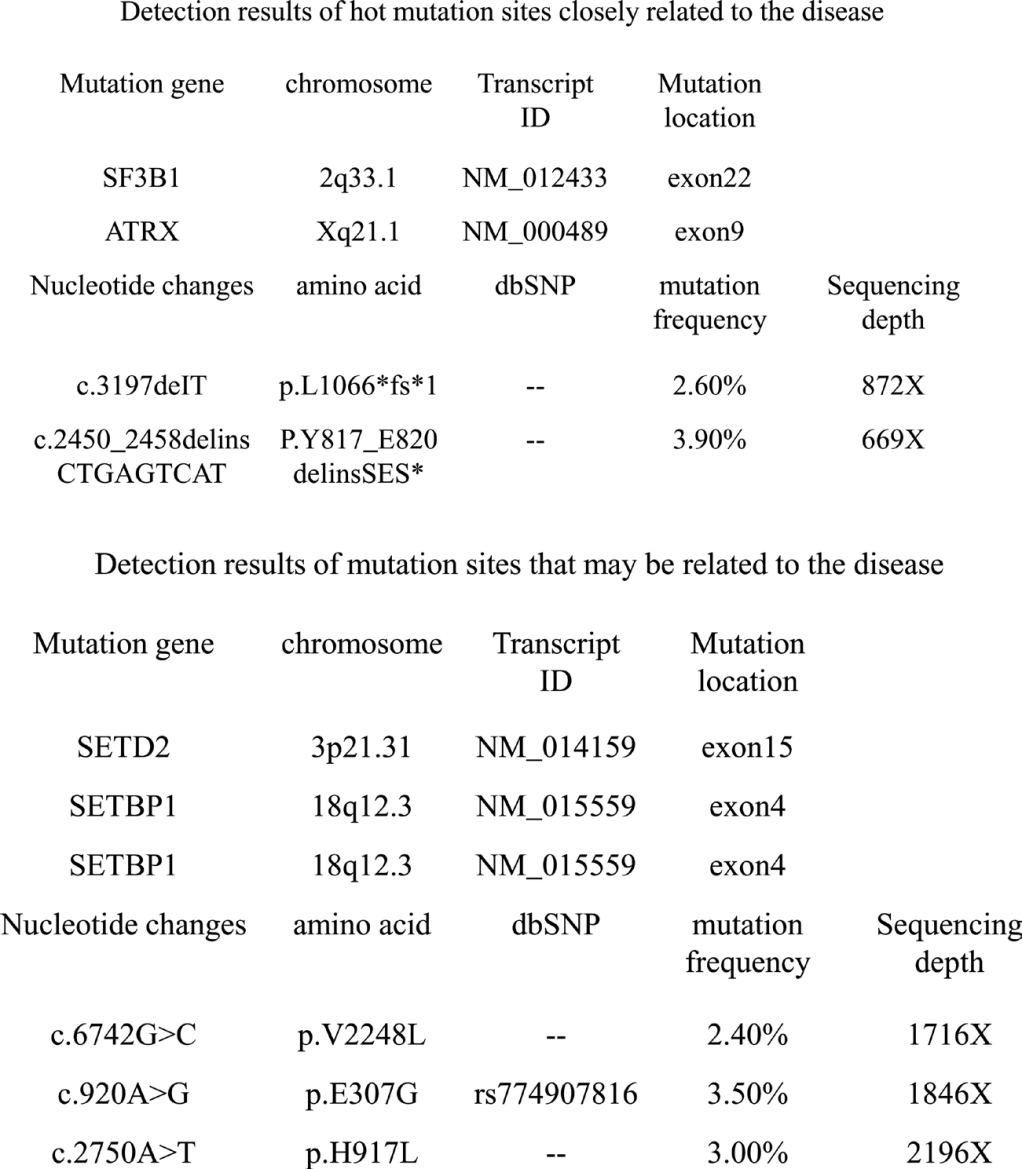
Gene screening of APL using second-generation sequencing. APL = acute promyelocytic leukaemia.

**Figure 6.**
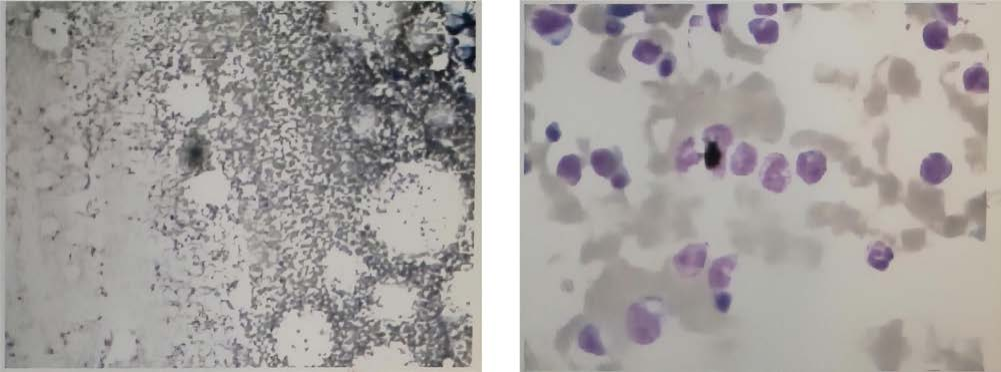
Chemical staining of bone marrow extract from patients after treatment.

**Figure 7.**
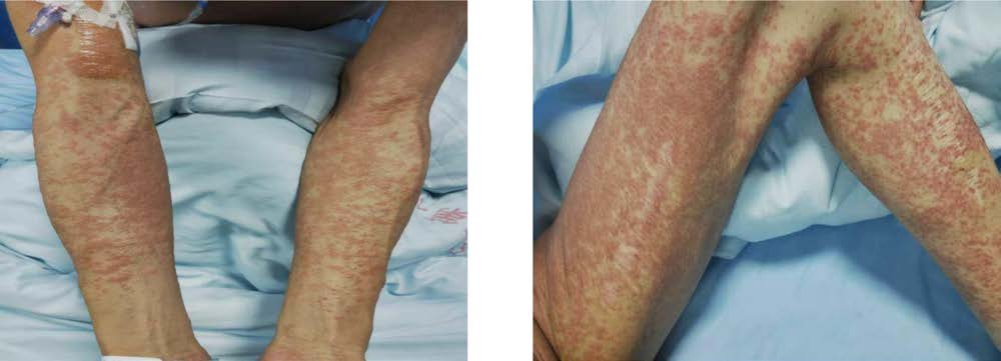
The patient presents with a skin appearance of red miliary papules.

**Figure 8.**
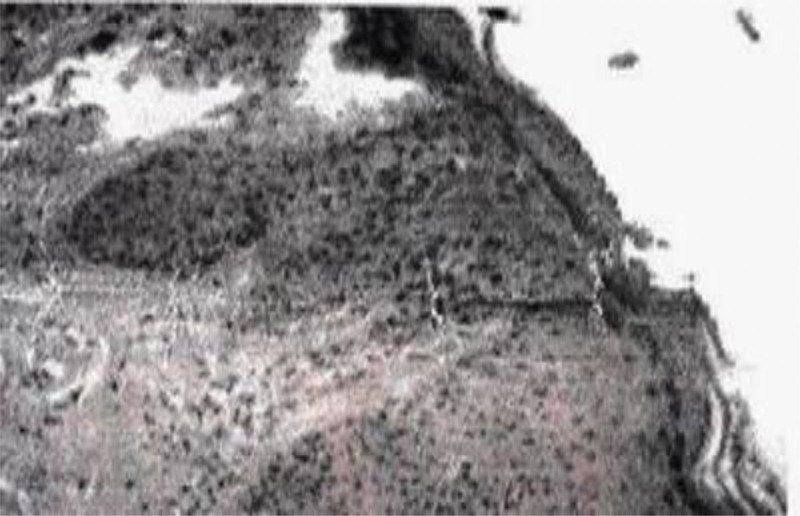
Pathological report of skin tissue biopsy.

The patient was hospitalized for pancytopaenia. After completing the examination, acute promyelocytic leukaemia was initially considered. He had no family genetic history. Previous physical health and physical examination showed no obvious abnormalities. The white blood cell count was 13 × 10^9/L, with a neutrophil ratio of 0.42 × 10^9/L, hemoglobin 122.0g/L, and platelets 59 × 10^9/L. Bone marrow morphology showed active bone marrow proliferation and obvious active granulocyte proliferation; promyelocytes comprised 82%, erythroid proliferation was reduced, the lymphocyte ratio was reduced, and megakaryocytes were not found in the whole film (Fig. 1). Flow cytometry of bone marrow aspirate showed positive results for CD33, MPO, CD117, CD38, CD13, and CD123 and negative results for C34, HLA-DR, TDT, and CD56. The abnormal cell population accounted for 86.3% of nuclear cells. This cases was consistent with the phenotype of APL (Fig. 2). Among 43 fusion genes, PML-RARA was positive (Fig. 3). Karyotype was 46, XY, (15:17) (q24; Q12) [16]/46, XY^[4]^ (Fig. 4). Second-generation sequencing showed that the SF3B1 mutation frequency was 2.6% and the ATRX mutation frequency was 3.9%; these are hot spot mutation sites closely related to the disease. The SETD2 mutation frequency was 2.4%, the SETBP1c.920A > G mutation frequency was 3.5%, and the SETBP1c.2750A > T mutation frequency was 3%, which may be related to the disease (Fig. 5). In conclusion, the patient was finally diagnosed with APL. There were no diagnostic challenges during our study. We administered tretinoin and arsenous acid (tretinoin 25 mg/m2, arsenous acid 0.16 mg/kg. d d1–12, daunorubicin 20 mg d6–10, 40 mg d11). Evaluation of the treatment effect on the 7th day after chemotherapy showed that the white blood cells and platelets of the patient had increased to normal levels (white blood cells 9.78 × 10^9/L, neutrophils 6.04 × 10^9/L, hemoglobin 104.0g/L, platelets 216 × 10^9/L); bone marrow morphology showed complete remission (Fig. 6). Minimal residual bone marrow was negative. The fusion gene PML-RARA was no longer detected. After the second course of chemotherapy, the patient developed red miliary macular papules (Fig. 7), which gradually worsened. Histopathological examination of the skin showed that the epidermis was hyperkeratotic, with incomplete keratosis. There was more lymphoid infiltration in the superficial layer of the dermis, and lymphoid cells had moved into the epidermis. The cells were immature, and early mycosis fungoides was possible. Immunohistochemistry of tumor cells showed CD2 (+), CD3 (+), CD4 (+), CD8 (partial +), CD20 (scattered +), CD30 (−), CD56 (−), TIA (partial +), GB (−), and Ki-67 (+) at approximately 10% (Fig. 8). Considering that the cases was complicated with skin mycosis fungoides, the patient was treated with budesonide ointment and methylprednisolone as chemotherapy. After 2 months of treatment, his condition gradually improved.

## 3. Discussion

Leukaemia is a group of malignant clonal diseases of hematopoietic cells that mainly manifest as symptoms caused by pancytopaenia and infiltration of leukaemia cells. Common infiltration sites of leukaemia are the liver, spleen, lymph nodes, central nerves and other sites; infiltration of the skin and mucosa is rare. Leukaemia can be divided into acute leukaemia and chronic leukaemia according to the degree of cell differentiation and maturity and the natural course of disease. The disease can be divided into lymphocytic leukaemia and nonlymphocytic (myeloid) leukaemia according to the cell series mainly involved. Acute promyelocytic leukaemia is a distinct subtype of acute myeloid leukaemia. The disease is characterized by a chromosomal abnormality involving translocation between chromosomes 15 and 17. Current therapy has advanced to include agents such as all-trans-retinoicacid and arsenic trioxide, which have improved remission and survival rates.^[4]^ During treatment, unique complications such as disseminated intravascular coagulation and differentiation syndrome or SS can occur. Therefore, prompt recognition of complications and initiation of appropriate treatment are imperative. MF and SS are the most common cutaneous T-cell lymphomas, a heterogeneous group of non-Hodgkin lymphomas of T-cell origin. MF is defined as primarily involving the skin. In the early stages, the disease typically presents in the form of erythaematous patches and/or plaques, which can persist for many years without clinical progression. In contrast, SS is characterized by fever and neutrophilia in addition to multiple painful asymmetrical erythaema patches.^[5,6]^

We report a patient with APL who developed multiple erythaematous skin lesions during ATRA treatment. The patient had no obvious fever, and no source of infection was found after extensive evaluation. Further progression of the patient’s condition did not occur after improvement of bone marrow and other related examinations. Considering that the patient developed erythaematous skin lesions after 2 courses of treatment with all-trans-retinoicacid, we performed skin biopsy and immunohistochemistry and finally determined it as early mycosis fungoides. In general, differentiation syndrome and SS caused by use of all-trans-retinoicacid are relatively common, but rare in the early stage of MF. Early intervention at this stage can greatly improve the prognosis and cure rate. Skin lesions are rapidly reduced by introduction of steroid therapy. Therefore, the side effects and complications caused by the application of ATRA in the treatment of APL must be given attention because mycosis fungoides should not only be distinguished from infectious diseases but also be further assessed with regard to disease progression and metastasis. For this patient, we distinguish it from infectious diseases, and actively make a clear diagnosis according to its skin performance. In response to this situation, we also promptly provided corresponding treatment plans to control the further development of the disease. The only downside is the failure to continue follow-up with patients, whether the patient’s disease is gradually improving or progressing, and the inability to further track it well. From this case, we further realize that due to the different sensitivities of different patients to drugs, it is necessary to pay attention to the drug dosage, potential adverse reactions during drug application, and potential problems with combination therapy during the medication process, make timely judgments, and actively improve the examination. Early diagnosis can guide accurate treatment, which is of great help in alleviating the pain of patients and improving the cure rate.

## Acknowledgements

We are very grateful to the teachers of Jining NO.1 People’s Hospital and Jining Medical College for their support and Mr. Wang’s cooperation.

## Author contributions

**Data curation:** Shasha Dong.

**Project administration:** Hongjing Zhou.

**Resources:** Fang Zhang.

**Writing – original draft:** Yongqin Zhao.

**Writing – review & editing:** Shasha Dong, Hongjing Zhou.
